# TERT mediates the U‐shape of glucocorticoids effects in modulation of hippocampal neural stem cells and associated brain function

**DOI:** 10.1111/cns.14577

**Published:** 2024-02-07

**Authors:** Meng‐Ying Liu, Yixin Fan, Ningjie Ni, Tao Yu, Zhiyuan Mao, Hanyu Huang, Jing Zhang, Yulin Tang, Hongliang He, Fan Meng, Yongping You, Qi‐Gang Zhou

**Affiliations:** ^1^ Department of Pharmacy, Nanjing Drum Tower Hospital Affiliated Hospital of Medical School, Nanjing University Nanjing China; ^2^ State Key Laboratory of Reproductive Medicine, Department of Clinical Pharmacology, School of Pharmacy Nanjing Medical University Nanjing China; ^3^ Department of Pharmacy, Sir Run Run Hospital Nanjing Medical University Nanjing China; ^4^ Department of Neurosurgery The First Affiliated Hospital of Nanjing Medical University Nanjing China; ^5^ School of Pharmacy Nanjing Medical University Nanjing China; ^6^ Key Laboratory for Aging & Disease, The State Key Laboratory of Reproductive Medicine, Department of Human Anatomy, Research Centre for Bone and Stem Cells Nanjing Medical University Nanjing China; ^7^ Department of Clinical Pharmacology, School of Pharmacy Nanjing Medical University Nanjing China

**Keywords:** bidirectional regulation, depression, glucocorticoids, learning and memory, NSCs, TERT

## Abstract

**Background:**

Glucocorticoids (GCs) are steroidal hormones produced by the adrenal cortex. A physiological‐level GCs have a crucial function in maintaining many cognitive processes, like cognition, memory, and mood, however, both insufficient and excessive GCs impair these functions. Although this phenomenon could be explained by the U‐shape of GC effects, the underlying mechanisms are still not clear. Therefore, understanding the underlying mechanisms of GCs may provide insight into the treatments for cognitive and mood‐related disorders.

**Methods:**

Consecutive administration of corticosterone (CORT, 10 mg/kg, i.g.) proceeded for 28 days to mimic excessive GCs condition. Adrenalectomy (ADX) surgery was performed to ablate endogenous GCs in mice. Microinjection of 1 μL of Ad‐mTERT‐GFP virus into mouse hippocampus dentate gyrus (DG) and behavioral alterations in mice were observed 4 weeks later.

**Results:**

Different concentrations of GCs were shown to affect the cell growth and development of neural stem cells (NSCs) in a U‐shaped manner. The physiological level of GCs (0.01 μM) promoted NSC proliferation in vitro, while the stress level of GCs (10 μM) inhibited it. The glucocorticoid synthesis blocker metyrapone (100 mg/kg, i.p.) and ADX surgery both decreased the quantity and morphological development of doublecortin (DCX)‐positive immature cells in the DG. The physiological level of GCs activated mineralocorticoid receptor and then promoted the production of telomerase reverse transcriptase (TERT); in contrast, the stress level of GCs activated glucocorticoid receptor and then reduced the expression of TERT. Overexpression of TERT by AD‐mTERT‐GFP reversed both chronic stresses‐ and ADX‐induced deficiency of TERT and the proliferation and development of NSCs, chronic stresses‐associated depressive symptoms, and ADX‐associated learning and memory impairment.

**Conclusion:**

The bidirectional regulation of TERT by different GCs concentrations is a key mechanism mediating the U‐shape of GC effects in modulation of hippocampal NSCs and associated brain function. Replenishment of TERT could be a common treatment strategy for GC dysfunction‐associated diseases.

## Graphical abstract

Schematic indicating that the U‐shaped effects of glucocorticoids (GCs) are mainly dependent on telomerase reverse transcriptase (TERT). Physiological levels of GCs can activate mineralocorticoid receptor, induce the upregulation of TERT, and ameliorate depressive‐like behavior in mice; in contrast, stressful levels of GCs can activate glucocorticoid receptor, reduce the levels of TERT, and induce depressive symptoms in mice.

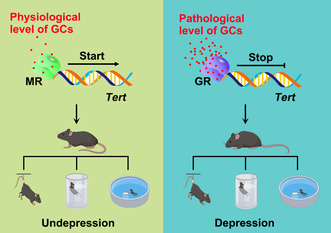

## 1 INTRODUCTION

Neurogenesis is a course of generating operational nerve cells from progenitor cells, which is conventionally viewed as happening exclusively during the embryonic and pre‐birth periods in mammals. However, Altman's research in the 1960s first reported the existence of newborn cells in the hippocampal dentate gyrus (DG) of rat brain.^1^ Goldman et al.'s study also confirmed the presence of neural regeneration in the ventricles of mature female canary brains.^2^ As the phylogenetic tree progressed, the number of widespread adult neurogenesis decreased, and neurogenic areas also became more defined, demonstrating the evolutionarily preserved adaptability of these particular areas. In rodents, adult neural regeneration primarily occurs in two areas of the brain. The first main neurogenic region is the hippocampal DG subgranular area.^3^ The next primary neuron‐generating area is the subventricular area of the lateral ventricles. In this region, newborn cells will move toward the olfactory bulb and act as interneurons.^4^, ^5^ Hippocampus is a highly plastic region of the mammalian brain, performing a vital function in episodic and locational memory.^6^ The newborn cells in the hippocampus DG communicate with others by integrating into neural circuits^7^, ^8^ and then increases the DG synaptic plasticity, which is essential in memory management.^9^, ^10^

Telomerase is a ribonucleoprotein utilizing RNA as a template to create telomeric repeats and add them to the ends of chromosomes to preserve telomere length^11^ and prevent chromosome fusion and wear,^12^ thereby preventing cell aging and apoptosis.^13^

Telomerase comprises a telomerase reverse transcriptase (TERT) and an RNA component holding a template for de novo synthesis of telomeres.^14^ Apart from its primary role in elongating and preserving telomeres, telomerase also is crucial for neuronal cells.^15^ Research has shown that TERT can migrate from the nucleus to the mitochondria in response to oxidative stress, leading to reduced reactive oxygen species (ROS), DNA damage, and apoptosis.^16^ Additionally, TERT also prevents Tau‐mediated pathological damage by reducing ROS in animal model of Alzheimer's disease.^15^ Additionally, shortening telomere length and reducing telomerase activity severely impair neuronal regeneration and differentiation.^17^ Our earlier research revealed that hippocampal TERT was involved in modulating depression‐like behaviors by regulating adult hippocampal neurogenesis^18^ and knockdown of TERT in DG impaired locational memory functions, while overexpression reversed these phenomena.^19^

Glucocorticoids (GCs) are steroidal hormones released by the fascicular zone of the suprarenal cortex, which regulate the biosynthesis and metabolism of carbohydrates, fats, and proteins. Stress can pathologically raise circulation levels of GCs by activating the hypothalamic–pituitary–adrenal axis,^20^ causing serial negative impacts, including impaired learning processes, memory function, and neural plasticity.^21^ Moderate levels of GCs or a mild stressor enhance long‐term potentiation (LTP) and improve spatial memory.^22^, ^23^ Conversely, high levels of GCs or chronic stress reduce LTP, promote long‐term depression (LTD), and impair spatial memory^24^, ^25^ with an inverted U‐shaped effect.^26^ Therefore, as conditions transition from stimulating to stress inducing, GCs transition from advantageous to detrimental.^27^ Although the strong appetency mineralocorticoid receptor (MR) and weak appetency glucocorticoid receptor (GR), which function as positive and negative modulators, respectively, are responsible for the inverted U‐shaped effect, the underlying mechanisms remain obscure.^28^, ^29^

Prior investigations have consistently pointed out that stress and activation of the GR could decrease hippocampal neurogenesis.^30^, ^31^ Selective knockdown of GR in hippocampal newborn neurons could accelerate its differentiation and migration. However, compared to GR, there have been contrasting effects regarding the role of MR in neurogenesis. Evidence indicates that MR knockdown could result in hindered neurogenesis and granular neuron deterioration in the mature hippocampal region.^32^, ^33^, ^34^, ^35^ Furthermore, studies have also found that injecting corticosterone (CORT) into bird embryos not only enhances telomerase activity but also prolongs the length of their telomeres after hatching.^36^ In this study, we primarily investigated the reciprocal regulation between glucocorticoids and telomerase, as well as their effects on neurogenesis, cognition, and memory.

## 2 MATERIALS AND METHODS

### 2.1 Animals and ethics

Eight‐week‐old, adult male C57BL/6J mice weighing between 18 and 22 g were acquired from Nanjing Medical University's Animal Core Facility. A cage containing four to five mice was maintained free to eat in a pathogen‐free environment with a temperature of 22 ± 2°C. All experimental protocols were performed following the standards guidelines of the National Institutes of Health (NIH) and received approval from the Experimental Animal Welfare Ethics Committee, Nanjing Medical University (No. IACUC‐2202017).

### 2.2 Chemicals and reagents

Corticosterone (#50–22‐6); 5‐bromo‐2‐deoxyuridine (BrdU, #B2531); metyrapone (#54–36‐4); mifepristone (#84371–65‐3); spironolactone (#52–01‐7); and dimethyl sulfoxide (DMSO, #67–68‐5) were obtained from Sigma‐Aldrich. The antibodies such as Ki67 (#9449), Nestin (#10959), and GAPDH (#2118) were obtained from Cell Signaling Technology; DCX (#ab18723) was acquired from Abcam; TERT (#bs‐0233R) was acquired from Bioss company; GR (#sc‐393,232) and MR (#sc‐53000) were obtained from Santa Cruz Biotechnology; and Hoechst (#23491–45‐4) was acquired from Sigma‐Aldrich. Neurobasal medium (#21103–049) and B27 (#12587–010) were acquired from Gibco. Epidermal growth factor (EGF, #AF‐100‐15‐100) and basic fibroblast growth factor (bFGF, #100‐18B‐100UG) were acquired from PeproTech and other cell cultivation reagents were acquired from Gibco. The Apoptosis Detection Kit (#556547) was acquired from BD (Biosciences).

### 2.3 Cell proliferation

Adult neural stem cells (NSCs) were cultured according to previously established methods.^37^, ^38^ The quantity of BrdU+ cells and NSCs neurospheres was used to measure cell proliferation in monolayers and neurospheres, respectively. Neurospheres were prepared into individual cells and then planted into 24‐well plates, 20,000 cells per well. Different concentrations of CORT (0.01, 0.1, 1, and 10 μM) were added to the cells when seeding. After 72 h, the neurospheres produced within every individual well were separated into a suspension of individual cells and the cell count was determined using a hemocytometer. For BrdU incorporation experiments, monolayers of NSCs were cultured on glass coverslips (2 × 2 cm) covered with polyornithine and laminin, and the cell proliferation test was performed following the manufacturer's guidelines.^19^

### 2.4 Adenovirus production and treatment

To create pDC315‐mTERT‐GFP, the DNA segments amplified by PCR and the pDC315‐green fluorescent protein vector were enzymatically cleaved with EcoR I and were joined with T4 DNA enzyme. In order to create the recombinant adenovirus (Ad‐mTERT‐GFP), HEK293 cells were simultaneously transfected with 5 μg of the pDC315‐GFP vector containing a complementary DNA sequence for mTERT and 5 μg of the pBHG lox E1,3 Cre vector as an auxiliary vector; collected the filtered HEK293 cells supernatant and utilized the Virus Purification Kit to purify the viral particles, and finally, detected the virus titer (2.5 × 10^10^ pFU/mL). Stereotaxic microinjection was used to deliver Ad‐mTERT‐GFP into the hippocampal DG at 2 μL per mouse. Ad‐GFP was used as control virus 4 weeks afterward to investigate the mice' behavior.

### 2.5 Flow cytometry

The Apoptosis Detection Kit I was employed for identifying cells undergoing apoptosis based on the guidelines provided by the manufacturer. In brief, 1 × 10^6^ NSC cells were cultured in six‐well plates. CORT was given to the cells for 72 h. FITC‐conjugated Annexin V and propidium iodide were added into the cells, softly swirling the cell mixture, and incubating at room temperature away from light for a while. In the final step, 400 μL of binding buffer was added to every respective tube to examine it using flow cytometry.

### 2.6 Stereotaxic injection

The operation was conducted according to previously established methods.^38^ The mice were anesthetized with isoflurane, and then secured in a computer‐controlled precision brain positioning device. Precision‐guided neurosurgery was carried out to administer the viral particles or liquid mixture into the hippocampus at a speed of 0.1 μL per minute. The spatial parameters are as follows: AP = −2.3 mm; ML = 1.35 mm; and DV = 2.3 mm. A warming mat was utilized for preserving body warmth, and ointment for eyes containing erythromycin was administered to the ocular area to avert dryness of the cornea. Mice that experienced bleeding post‐needle withdrawal were omitted from the experiments.

### 2.7 The Morris water maze (MWM) test

The MWM test was conducted following previously outlined methods with a few adjustments.^19^ The visible platform variant of MWM's training paradigm included four trials (the maximum time is 60 s and the interval time is 15 min) daily for 2 successive days. Then, the concealed platform variant of the MWM test was performed in the following 5 days. The test trial was conducted 24 h subsequent to the training period concluded on the 6th day. The swimming trajectories were captured using a system for tracking movements using video technology (EthoVision XT). The mice that remained afloat or leaped off the platform throughout the duration of the test were excluded.

### 2.8 Tail suspension test (TST)

The TST was conducted in line with previously established methods, with some modifications.^39^ The mice were positioned in the new environment for 12 h to acclimatize to the unfamiliar surroundings. The mouse tail was adhered to adhesive tape at 1 cm from the end. The mouse was hung upon a soundproof box hanging rod and the height from the point where its tail tip and the ground intersect was approximately 30 cm, and the mouse was placed in a head‐inverted position. The total duration of the experiment was 6 min, the first 2 min was the time for the mice to adapt to the suspension, and the duration of inactivity in the mice during the final 4 min was recorded.

### 2.9 Forced swimming test (FST)

Forced swimming test was executed following earlier outlined procedures with minor modifications.^39^ Mice swam in water at 25°C for about 5–10 min the day before the test. The following day, mice were separately compelled to swim in an open cylindrical glass container. The complete duration of motionlessness was documented throughout a 6‐min examination. Mouse was recognized as motionless when it stopped making any effort and stayed afloat without any movement in the water, just maintaining its head over the water's level. Water was refreshed after testing five mice. After swimming sessions, the mice were dried using a towel and exposed to a heat lamp for 30 min before being returned to the cage.

### 2.10 Sucrose preference test (SPT)

Sucrose preference test was carried out following the previously provided description.^40^ In brief, mice were housed in a single cage with two containers of 1% sucrose solution, and one of the bottles was changed to pure water after 24 h. After training, mice were water‐deprived for 24 h and then subjected to a SPT. Mice were allowed to consume the fluids for 24 h. After that, the bottles were taken out and weighed. The percentage of sucrose consumption = sucrose solution intake/(sucrose solution intake + pure water intake) × 100%.

### 2.11 Fluoro‐Jade staining

Fluoro‐Jade staining was employed for identifying neuronal loss and degeneration.^18^, ^19^ The sections of the brain were transferred to glass microscope slides and then immersed in solution of absolute ethanol for a duration of 3 min, a 70% ethanol solution for 1 min, and pure distilled water for 1 min. The glass slides were then placed into a solution containing 0.01% Fluoro‐Jade and 0.1% acetic acid for a duration of 30 min while being lightly shaken, and then rinsed with 1x PBS three times, and finally, cover slipped and analyzed using confocal microscopy.

### 2.12 Middle cerebral artery occlusion (MCAO)

Localized brain ischemia was triggered by MCAO, as explained earlier.^18^, ^19^ The mice were rendered unconscious using 1% sodium pentobarbital and fixed on the operating table. After disinfection of the neck, the internal, external, and common carotid blood vessels were exposed, the common and outer carotid arteries were tied off, and the tether was gradually introduced into the left interior carotid artery through the remaining part of the outer carotid artery until a minor level of resistance was felt. After 1.5 h, the tether was removed. During the operation, the body temperature was upheld at 37 ± 0.5°C. In the sham‐operated mode, the blocking link was introduced 7 mm above the point where the carotid artery divides into two branches.

### 2.13 Western blotting

Collected the NSC samples and lysed in a radioimmunoprecipitation assay cell lysis solution. Protein levels were assessed using BCA Protein Assay, and 40 μg of protein was loaded and fractionated via SDS‐PAGE, electrophoretically moved to polyvinylidene fluoride (PVDF) membranes, and then obstructed using 5% bovine serum albumin for a duration of 2 h at room temperature and subjected to overnight antibody probing at 4°C using primary antibodies, After that, secondary antibodies conjugated with horseradish peroxidase (HRP) were incubated. The primary antibodies employed were TERT (1:1000), MR (1:1000), GR (1:1000), and Nestin (1:1000). As a control for protein loading, the blots were examined using antibodies targeting GAPDH (1:1000). The signals were then detected by enhanced chemiluminescence. Data were collected and analyzed using ImageJ software. The oringnial bands of the representative WB results are provided in the Supplemental File Data S1.

### 2.14 Immunohistochemistry

Mice were anesthetized with isoflurane and underwent transcardial perfusion using saline solution, followed by 4% paraformaldehyde (PFA). Collected the brain and continued to fix it in 4% PFA for one night. Consecutive sections were sectioned as 40 μm thick. The samples were incubated with rabbit antibodies against Ki67 (1:200) for 16 h at 4°C. The free‐floating sections were rinsed with PBS three times before undergoing incubation with secondary antibodies (1:300) at room temperature. For celling staining, NSC cells were treated with 4% PFA for fixation and were permeabilized using 0.3% Triton X‐100. The samples were blocked with 10% serum from donkeys for 2 h and then incubated with MR (1:200) or GR (1:200) at 4°C for 16 h. After being incubated for 2 h at room temperature with fluorescent secondary antibodies (1:300), the coverslips were rinsed three times with PBS. Hoechst is a type of fluorescent dye used to label the nucleus. Imaging was captured by Carl Zeiss LSM880 confocal system.

### 2.15 Examination of neuronal spine density and morphology

Pictures of neurons labeled with RFP or DCX^+^ neurons were captured by the confocal microscope (Carl Zeiss LSM880 confocal system), and the Imaris v7.2.3 software was used to reconstruct the morphology. The measurement of each dendritic segment's length was calculated by tracing the central line of the portion of the dendrite, and the number of spines was tabulated by manual inspection from the 2D representations. The straight spine density was computed by dividing the overall count of spines by the length of the specific portion of the dendrite. Confocal microscopy imaging and quantification of data were both conducted by the same individual, who was unaware of the experimental variables.

### 2.16 Statistical analyses

Statistical tests were carried out by GraphPad Prism 10 (GraphPad Software). All the data were subjected to testing for their normal distribution using the Kolmogorov–Smirnov (K‐S) test method. Following a homogeneity test for variance, when it was assumed that variances were equal, unpaired or paired Student's *t* test was employed to assess the disparities between two groups, and one‐way ANOVA was utilized for making comparisons among three or four groups. Results are presented as mean ± SEM. Data that are described as significant are determined based on a criterion of *p* < 0.05.

## 3 RESULTS

### 3.1 GCs bidirectionally modulate NSC proliferation in vitro

**FIGURE 1:**
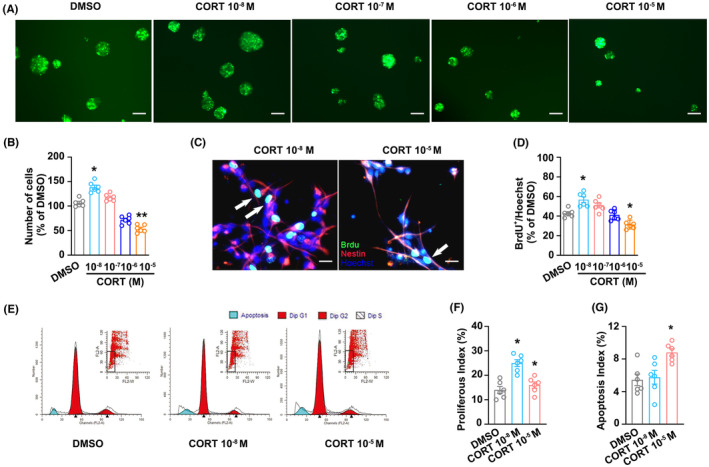
GCs bidirectionally modulate proliferation of NSCs in vitro. (A) Adult NSC cells were treated with different concentrations of CORT (0.01, 0.1, 1, and 10 μM) or 0.1% DMSO for 72 h, and then the size of adult NSCs neurospheres was detected. (B) Statistical graph from the data in (A). (C) Representative images of BrdU‐labeled cells (green) of the monolayer‐cultured adult NSCs (red) treated with physiological levels of GCs (0.01 μM) and stressful levels of GCs (10 μM). Hoechst (blue) is labeled nucleus. (D) Statistical graph showed the number of BrdU‐labeled cells of the monolayer‐cultured adult NSCs treated with different concentrations of CORT (0.01, 0.1, 1, and 10 μM) or 0.1% DMSO for 72 h. (E–G) Representative images and statistical graphs showed the proliferous and apoptosis index assessed by flow cytometric analysis. Magnification: 100×, 200×. Scale bar: 20 μm. Significant differences were obtained using one‐way or two‐way ANOVA (*p < 0.05, **p < 0.01 vs. DMSO group; Bonferroni post‐hoc tests). CORT, corticosterone; DMSO, dimethyl sulfoxide; GCs, glucocorticoids; NSCs, neural stem cells.

To determine the effect of GCs on NSCs in vitro, NSCs were subjected to varying concentrations of CORT (0.01, 0.1, 1, and 10 μM) for 72 h. The findings indicated that the stressful level of GCs (10 μM) reduced the formation of neurospheres, while the size of NSCs neurospheres was increased by the physiological level of GCs (0.01 μM) compared with the DMSO group (Figure 1A,B). Comparable outcomes were observed in BrdU incorporation experiments conducted on monolayer‐cultured NSCs. The physiological level of GCs (0.01 μM) significantly increased, but the stressful level of GCs (10 μM) decreased cell proliferation (Figure 1C,D). Moreover, flow cytometry obtained similar results. The physiological level of GCs (0.01 μM) promoted NSC cells proliferation in vitro, while the stressful level of GCs (10 μM) reduced these effects (Figure 1E,F). Additionally, the stressful level of GCs (10 μM), rather than physiological level of GCs (0.01 μM), mediated the apoptosis of NSCs in vitro (Figure 1E,G). Taken together, these results indicate that GCs bidirectionally modulate NSCs' proliferation in vitro.

### 3.2 GCs bidirectionally modulate adult hippocampal neurogenesis

**FIGURE 2:**
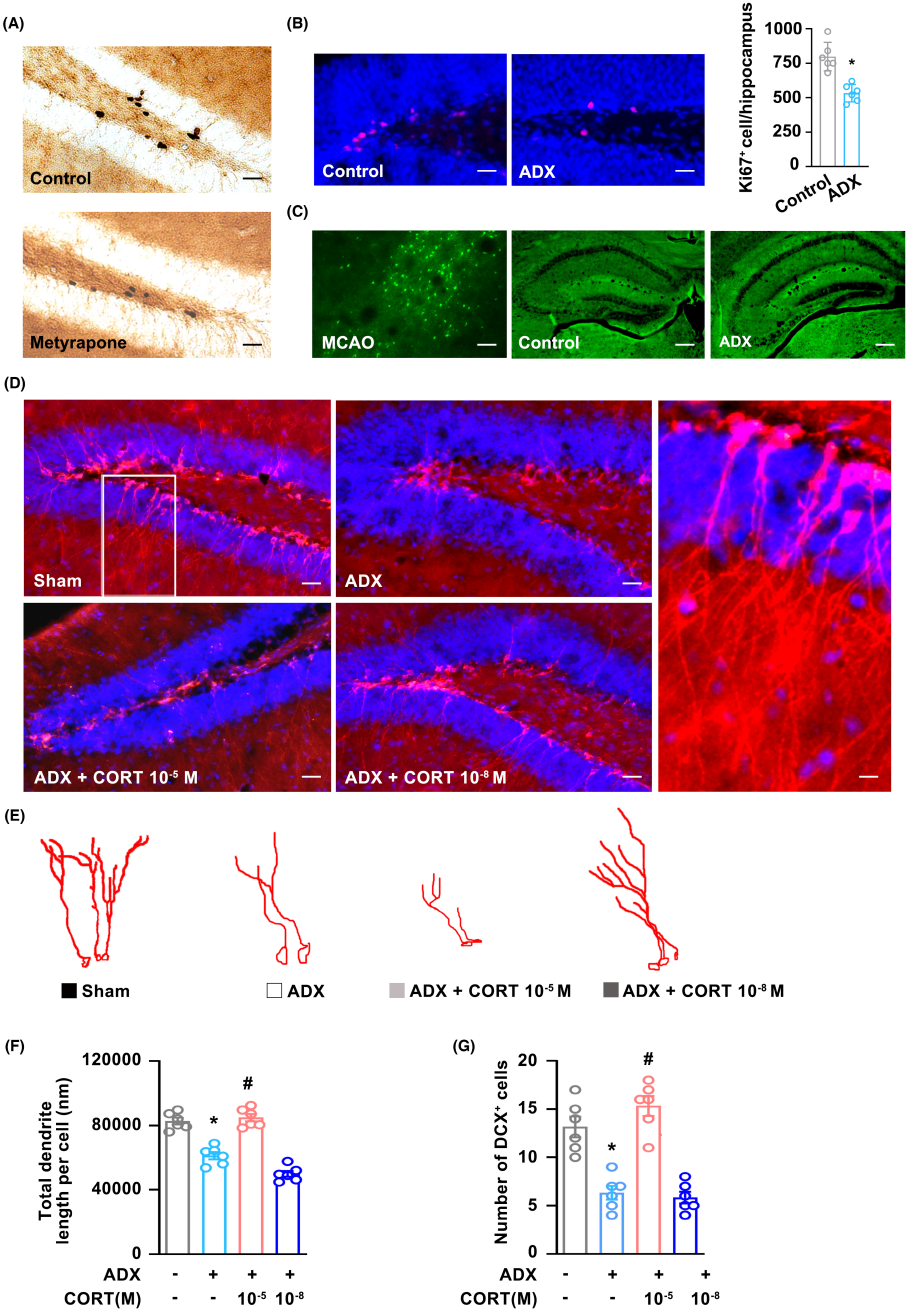
GCs bidirectionally modulate adult hippocampal neurogenesis. (A) Representative images showed BrdU‐positive cells in the adult hippocampal DG at 24 h after metyrapone treatment in mice. (B) Representative images and statistical graphs showed Ki67‐positive cells in the adult hippocampal DG at 3 days after ADX treatment in mice. (C) Representative images showed no FJ‐positive cells located in the adult hippocampal DG at 3 days after ADX treatment, but lots of FJ‐positive cells in MCAO‐exposed mice. (D) Representative immunostaining images showed that physiological levels of GCs (0.01 μM) could rescue ADX‐mediated reduction in DCX^+^ immature neurons in the hippocampal DG, but not in stressful levels of GCs (10 μM). (E–G) Bar graph showing the number of DCX^+^ neurons and total dendritic length per DCX^+^ neuron at physiological and stressful levels of GCs in the hippocampal DG of ADX mice. Magnifications: 200×, 400×. Scale bar: 20 μm. Significant differences were obtained using one‐way or two‐way ANOVA (*p < 0.05 vs. vehicle group; ^#^ *p* < 0.05 vs. ADX treatment group; Bonferroni post‐hoc tests). ADX, adrenalectomy; DCX, DG, dentate gyrus; FJ, Fluoro‐Jade; GCs, glucocorticoids; MCAO, middle cerebral artery occlusion.

Mounting evidence has documented a strong reciprocal connection between GCs and neurogenesis in the hippocampus.^41^, ^42^, ^43^ Although reports documented that elevated CORT damages adult hippocampal neurogenesis and contributes to neuronal loss, studies also report that circulating GCs would affect the cell proliferation of the DG granule cell progenitors.^44^ This suggests that GCs may play different roles in adult hippocampal neurogenesis. In this study, we found that pretreatment with GCs inhibitor (metyrapone, 100 mg/kg, i.p.) significantly reduced adult hippocampal neurogenesis (Figure 2A). Besides, GC deprivation induced by adrenalectomy (ADX) also dramatically decreased cell proliferation in adult hippocampal DG (Figure 2B). Moreover, we further investigated whether removing the circulating GCs brought damage to hippocampal DG neurons. Mice were euthanized to assess cellular damage by Fluoro‐Jade (FJ) staining. It was found that there were no FJ‐labeled cells in ADX mice hippocampus (Figure 2C). Meanwhile, ischemia induced in the mouse brain via intraluminal MCAO was employed as a positive reference and lots of FJ‐positive cells were located in the MCAO group (Figure 2C). Additionally, in comparison with the sham group, we discovered that ADX dramatically decreased the quantity of DCX^+^ immature neurons (Figure 2D) as well as the total dendritic length of immature newly formed neurons in the hippocampal DG (Figure 2E‐G). However, pretreatment with physiological level of GCs (0.01 μM) reversed these effects, instead of the stressful level of GCs (10 μM; Figure 2E‐G). Therefore, these results demonstrated that GCs bidirectionally modulated adult hippocampal neurogenesis in mice.

### 3.3 Bidirectional regulation of telomerase by GCs mediates the U‐shaped effect on neurogenesis

**FIGURE 3:**
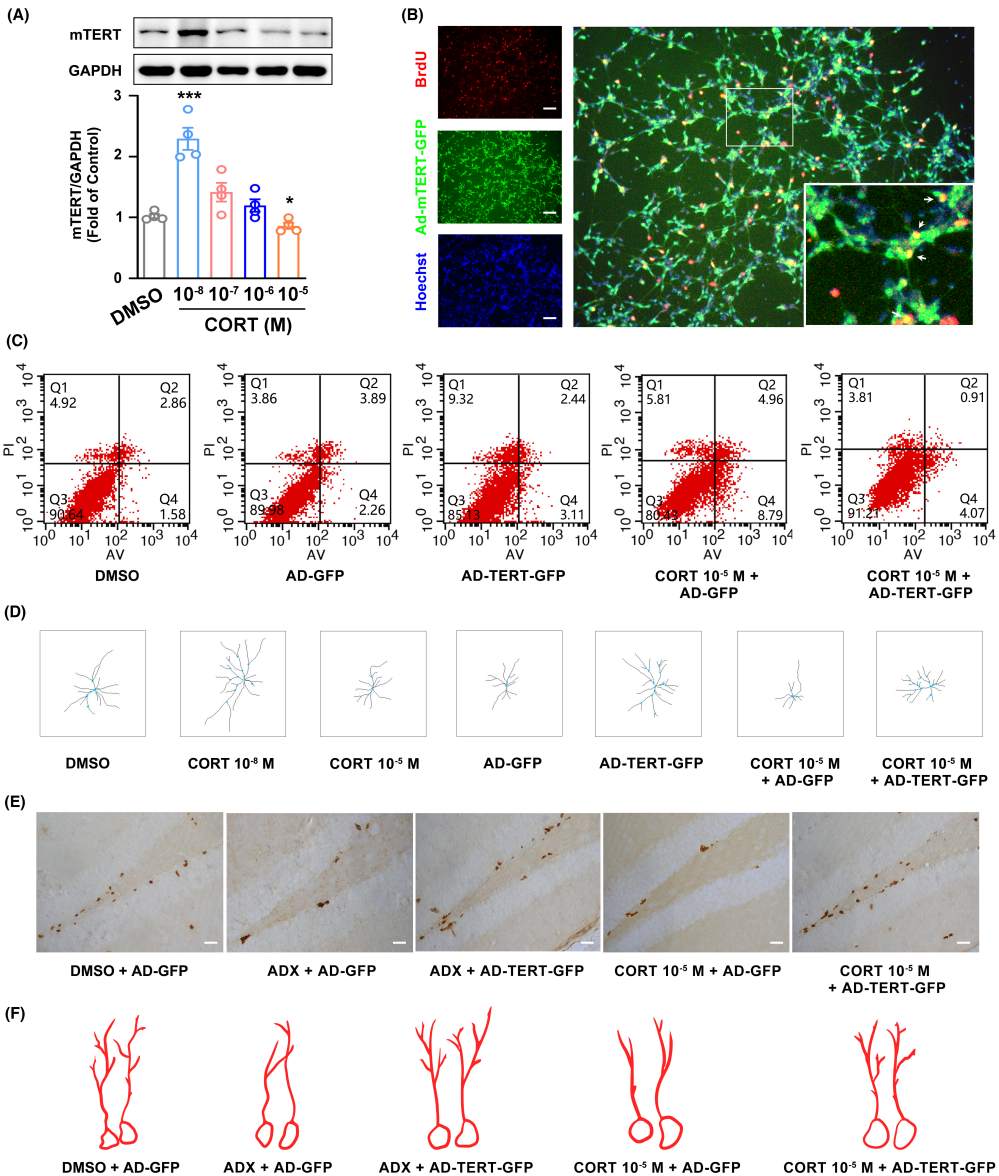
Bidirectional regulation of TERT expression by glucocorticoids (GCs) accounting for U‐shaped effect on neurogenesis. (A) Representative western blot images and statistical graphs showed the expression of mTERT in CORT‐treated cells in vitro. NSC cells were treated with different concentrations of CORT (0.01, 0.1, 1, and 10 μM) or 0.1% DMSO for 72 h. (B) Representative immunostaining images show that numerous GFP^+^ cells were colocalized with BrdU^+^ cells in vitro. The monolayer‐cultured NSCs were transfected with AD‐mTERT‐GFP (1 μL/wells, titer: 2.5 × 10^10^ pfu/mL) for 16 h, and then continued to culture for 72 h after changing the medium. (C) Flow cytometric images showed the apoptosis index of NSC cells in vitro. NSC cells were transfected with AD‐mTERT‐GFP or AD‐GFP for 16 h, and then cocultured with stressful levels of GCs (10 μM) for 72 h, Ad‐GFP was as control virus. (D) Bar graph showed the dendrites branching in CORT (0.01 or 10 μM) group and AD‐mTERT‐GFP group. Representative images showed that overexpression of mTERT induced by AD‐mTERT‐GFP could reverse the stressful levels of GCs (10 μM) or ADX induced by the reduction in Ki67‐positive cells (E), and the total length of dendrites of immature newborn neurons (F) in the hippocampal DG. Magnifications: 100×, 400×. Scale bar: 20 μm. Significant differences were obtained using one‐way or two‐way ANOVA (*p < 0.05, ***p < 0.001 vs. vehicle group; Bonferroni post‐hoc tests). ADX, adrenalectomy; CORT, corticosterone; DG, dentate gyrus; DMSO, dimethyl sulfoxide; GCs, glucocorticoids; NSCs, neural stem cells; TERT, telomerase reverse transcriptase.

To investigate whether GCs regulate the expression of TERT in NSC cells. NSCs cells were subjected to varying doses of CORT (0.01, 0.1, 1, and 10 μM) for 72 h. Western blot demonstrated that the physiological level of GCs (0.01 μM) upregulated TERT expression, while stressful level of GCs (10 μM) downregulated TERT expression (Figure 3A). To further investigate whether TERT could promote the formation of newborn cells, the monolayer‐cultured NSCs were transfected with AD‐mTERT‐GFP for 16 h, followed by BrdU staining after medium change and cell culture for 72 h. The confocal images showed that numerous GFP^+^ cells were colocalized with BrdU^+^ cells in vitro (Figure 3B). Moreover, we also found that NSC cells transfected with AD‐mTERT‐GFP did not cause apoptosis compared with the DMSO group (Figure 3C). On the contrary, AD‐mTERT‐GFP might play a role in the decrease in apoptosis under the stressful level of GCs (Figure 3C). AD‐mTERT‐GFP also significantly increased the branches of dendrites in immature newborn neurons, which was similar to the effect of the physiological level of GCs (0.01 μM; Figure 3D); furthermore, we also found that overexpression of TERT induced by AD‐mTERT‐GFP could reverse the reduction in dendrites branching mediated by the stressful level of GCs (10 μM; Figure 3D). Moreover, overexpression of TERT was capable of reversing GCs deprivation (ADX) or the stressful level of GCs induced the reduction in total length of dendrites of immature newly formed neurons in the adult DG (Figure 3F), as well as the quantity of Ki67^+^‐positive cells in vivo (Figure 3E). These findings suggested that GCs bidirectionally controlled the function of telomerase, and overexpression of TERT could significantly reverse abnormal GCs‐mediated reduction in newborn cells in vivo.

### 3.4 Mineralocorticoid receptor and GR differentially mediate the U‐shape of GC effects

**FIGURE 4:**
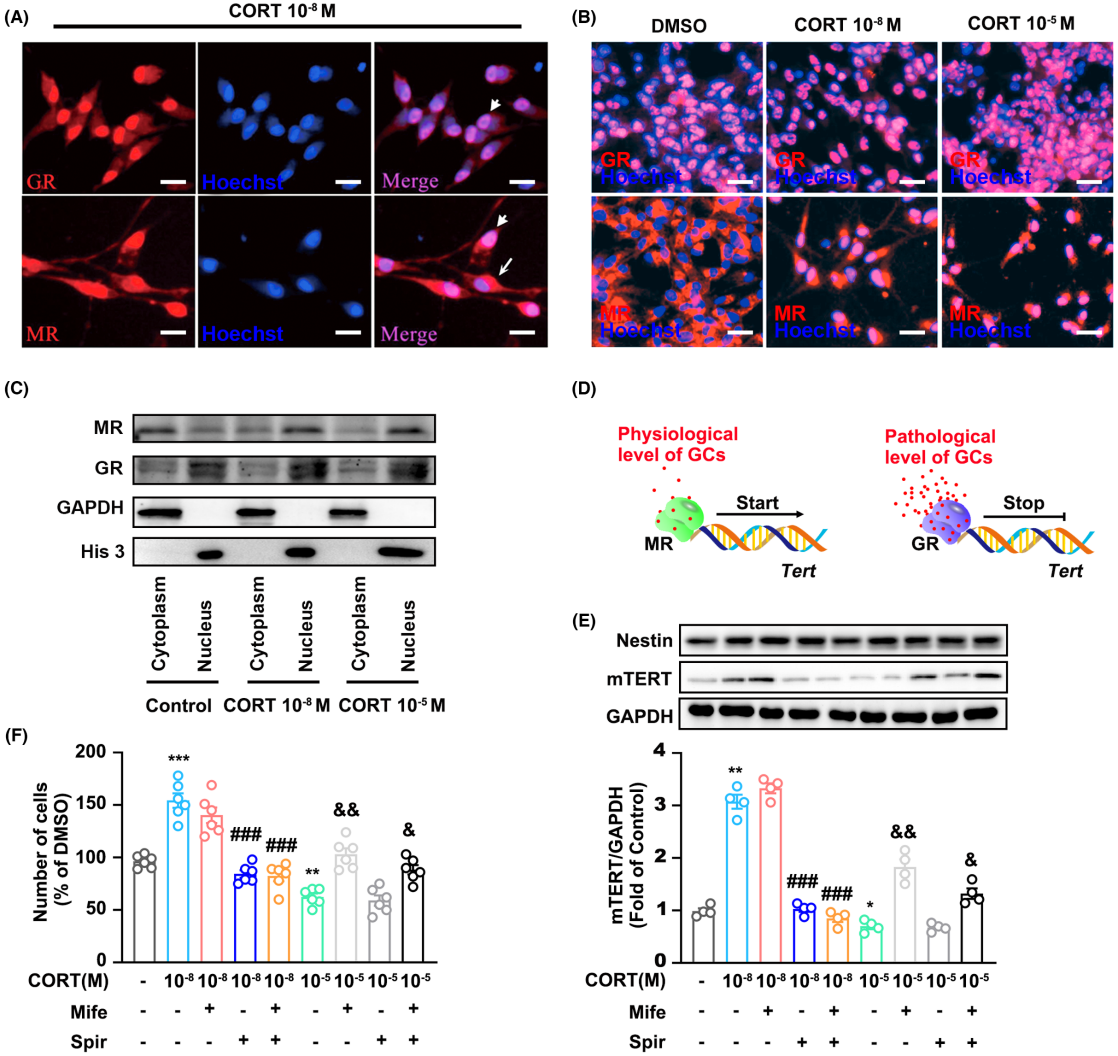
MR and GR differentially mediate the U‐shaped effect of GCs on TERT expression. (A, B) Representative immunostaining images showing the levels of MR or GR in the nucleus in monolayer‐cultured adult NSCs treated with physiological levels of GCs (0.01 μM), or stressful levels of GCs (10 μM) for 72 h. (C) Representative western blot images showing the expression of MR and GR in the cytoplasm or nucleus. (D) Schematic indicating that GCs at physiological levels or stressful levels regulate the expression of mTERT by activating MR or GR, respectively. (E) Representative western blot images and statistical graphs showing the expression of mTERT and Nestin in vitro. Pretreatment with MR antagonist (spironolactone, spir) or GR antagonist (mifepristone, mife) in NSCs cells for 30 min, and then coculture with physiological levels of GCs (0.01 μM) or stressful levels of GCs (10 μM) for 72 h, respectively. (F) Statistical graph showing the size of adult NSCs neurospheres in vitro. Magnifications: 200×. Scale bar: 20 μm. Significant differences were obtained using one‐way or two‐way ANOVA (*p < 0.05, **p < 0.01, ***p < 0.001 vs. vehicle group; ^###^ *p* < 0.001 vs. CORT (physiological levels) treatment group; and ^&^ *p* < 0.05, ^&&^ *p* < 0.01 vs. CORT (stressful levels) treatment group; Bonferroni post‐hoc tests). CORT, corticosterone; GCs, glucocorticoids; GR, glucocorticoid receptor; MR, mineralocorticoid receptor; NSCs, neural stem cells; TERT, telomerase reverse transcriptase.

The above data indicated that GCs could bidirectionally regulate adult hippocampal neurogenesis, and these effects were dependent on telomerase activity, but the fundamental mechanism remains unclear. Here, we observed that the physiological level of GCs (0.01 μM) significantly promoted MR translocation to the nucleus in most cells, while it could also induce GR translocated into the nucleus in a small proportion of cells (Figure 4A). In contrast, the stress levels of GCs (10 μM) could induce MR and GR movement to the nucleus in most cells (Figure 4A,B). Western blot also indicated that both the physiological and stressful levels of GCs significantly promoted MR translocation from cytosol to nucleus compared with DMSO (Figure 4C). However, as compared to the physiological level of GCs (0.01 μM), stressful level of GCs (10 μM) was more efficient in inducing GR translocation to nucleus (Figure 4C). This is consistent with earlier investigations. Whitehead et al.^45^ indicated that GR could inhibit the expression of CREB protein. These results suggested that the physiological level of GCs (0.01 μM) increased TERT protein expression mainly by activating MR, whereas the stressful level of GCs (10 μM) inhibited TERT expression by activating GR (Figure 4D). Moreover, we also found that MR antagonist (spironolactone, Spir) could significantly inhibit the upregulation of TERT protein mediated by the physiological level of GCs (0.01 μM), whereas GR antagonist (mifepristone, Mife) had no similar effect. Besides, combined application of Spir and Mife also significantly reduced the expression levels of TERT (Figure 4E). In contrast, Mife could reverse the downregulation of TERT protein mediated by the stressful level of GCs (10 μM), and combined with Spir showed similar effects as Mife alone, but Spir alone could not reverse this effect (Figure 4E). Furthermore, Spir could also reduce the size of NSCs neurospheres in the physiological level of GC (0.01 μM) group, while Mife could reverse the stressful level of GCs (10 μM) mediated by decrease in NSC size in vitro (Figure 4F).

### 3.5 Replenishment of TERT rescues GCs deficiency‐caused learning and memory decline and depression

**FIGURE 5:**
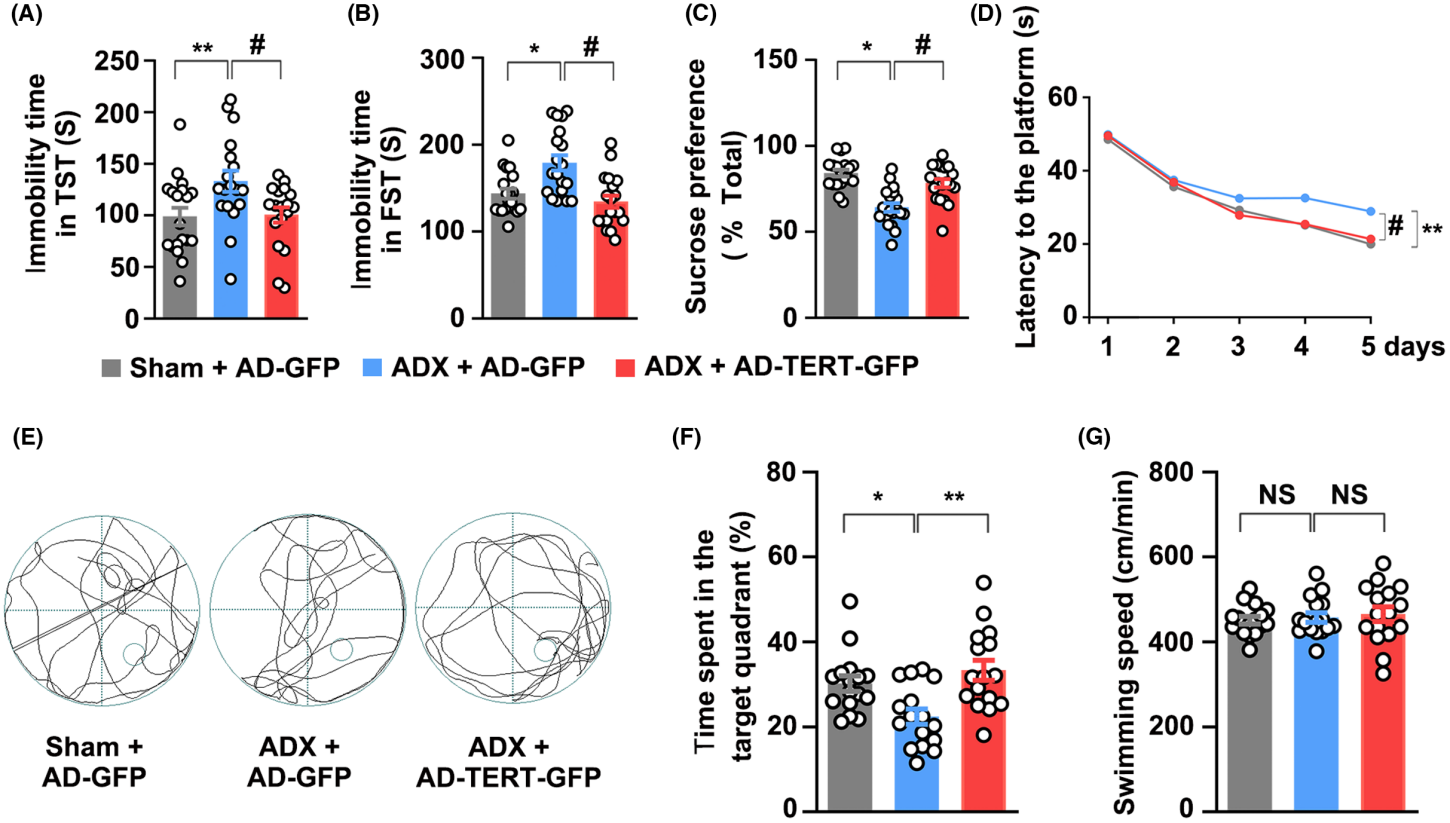
TERT re‐expression rescues GCs deficiency‐caused memory decline and depression. Microinjection of AD‐mTERT‐GFP virus into the hippocampal DG for 28 days significantly rescued depressive‐like behaviors in ADX‐treated mice in the TST (A), FST (B), and SPT (C). (D–F) Overexpression of mTERT induced by AD‐mTERT‐GFP could rescue spatial memory in ADX mice. Representative images of swimming paths in MWM task (D), (E) time spent in target quadrant, and escape latency (F). (G) The mice treated with ADX or AD‐mTERT‐GFP did not affect swimming speed. *N* = 20. Significant differences were obtained using one‐way or two‐way ANOVA (*p < 0.05, **p < 0.01 vs. vehicle group; ^#^ *p* < 0.05 vs. ADX treatment group; Bonferroni post‐hoc tests). ADX, adrenalectomy; DG, dentate gyrus; FST, forced swimming test; GCs, glucocorticoids; MWM, Morris water maze; SPT, sucrose preference test; TERT, telomerase reverse transcriptase; TST, tail suspension test.

As illustrated in Figure 5A‐C, in comparison to the sham group, GC deprivation mediated by ADX significantly increased the duration of immobility in TST and FST, and decreased the sucrose intake in mice. However, overexpression of TERT induced by AD‐mTERT‐GFP transfection markedly reversed ADX‐mediated increase in the duration of immobility in TST and FST and increased the sucrose preference in mice. In addition, compared with the sham group, MWM test showed that the latency to reach the platform was increased over 5 days of training in ADX mice, which indicated that GC deficiency might affect learning and memory. However, ADX mice transfected with AD‐mTERT‐GFP were able to quickly find the platform (Figure 5D,E) and remained longer in the target quadrant than ADX mice (Figure 5F). Additionally, both ADX and TERT overexpression did not affect the swimming speed of mice (Figure 5G). Altogether, these results showed that TERT overexpression could reverse the decline in learning and memory mediated by GC deficiency in ADX mice.

### 3.6 Replenishment of TERT rescues chronic stress‐caused learning and memory decline and depression

**FIGURE 6:**
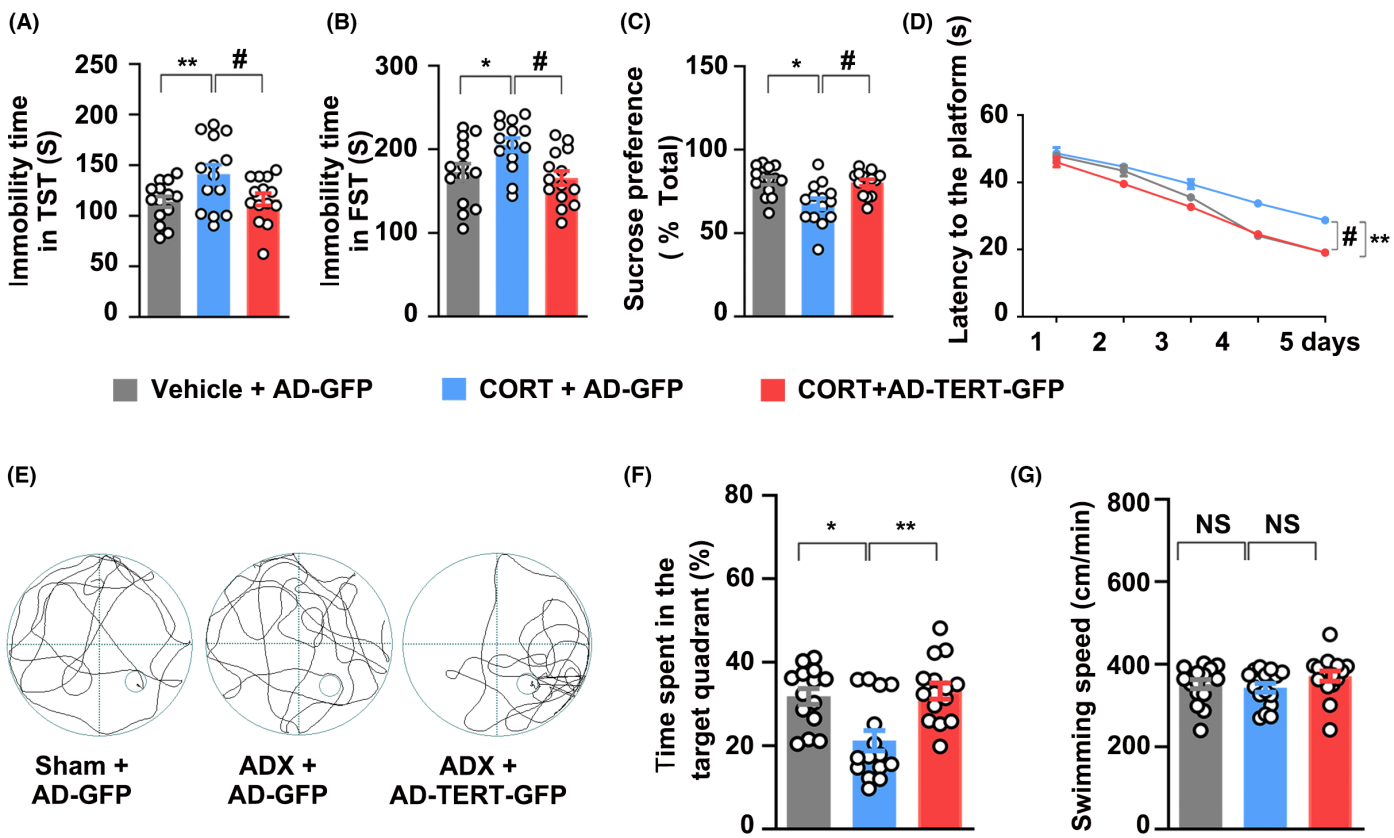
TERT re‐expression rescues high levels of GCs‐induced depression symptom and memory decline. Consecutive administration of CORT (10 mg/kg, i.g.) for 28 days significantly induced the formation of depressive‐like behaviors in mice. Immobility time in TST (A) and FST (B), and the sucrose preference test (SPT) (C). (D–F) Overexpression of mTERT induced by AD‐mTERT‐GFP could rescue spatial memory in COTR‐treated mice. Representative images of swimming paths in MWM assay (D), (E) time spent in the target quadrant, and escape latency (F). (G) The mice treated with CORT or AD‐mTERT‐GFP did not affect swimming speed. *N* = 20. Significant differences were obtained using one‐way or two‐way ANOVA (*p < 0.05, **p < 0.01 vs. vehicle group; ^#^ *p* < 0.05 vs. CORT treatment group; Bonferroni post‐hoc tests). CORT, corticosterone; FST, forced swimming test; GCs, glucocorticoids; SPT, sucrose preference test; TERT, telomerase reverse transcriptase; TST, tail suspension test.

The above data indicated that the stressful level of GCs suppresses adult hippocampal neurogenesis possibly through activating GR to inhibit TERT expression. In this study, in contrast to vehicle group, we found that successive application of 10 mg/kg of CORT into mice for 28 days increased the duration of immobility in TST and FST, and reduced the sucrose intake in mice, whereas overexpression of TERT induced by AD‐mTERT‐GFP reversed these effects (Figure 6A‐C). In MWM assay, the mice transfected with AD‐mTERT‐GFP reached the platform more quickly than the CORT group after training for 5 days (Figure 6D,E) and extended the time remaining in the target quadrant (Figure 6F) without affecting swimming speed (Figure 6G). These data suggested that TERT overexpression could ameliorate depression‐like behaviors in mice induced by high levels of CORT.

## 4 DISCUSSION

In this study, we discovered that the physiological and stress levels of GCs exhibit opposite effects while deficient and excessive GCs have similar effects on regulating NSC proliferation, adult hippocampal neurogenesis, TERT expression, learning and memory, and depression phenotype. Our data suggested that the U‐shaped effect of GCs is mainly dependent on the level of TERT in NSCs. Physiological level of GCs activated MR and upregulated TERT expression; in contrast, stressful level of GCs activated GR and decreased the levels of TERT. Moreover, overexpression of TERT could reverse the decline in learning and memory, as well as depressive symptoms, induced by high levels of GCs or GC deprivation in mice. Our findings have provided new insights into the bidirectional mechanism of GCs in neuropsychiatric diseases, including memory impairment and depressive disorder.

As a hormone released by the adrenal cortex under stressful situations, GCs are not only essential for the development, growth, metabolism, and immunity but also critical for numerous brain functions, including arousal, sleep, behavior, cognition, memory, mood, affect, etc.^46^, ^47^ Studies have also indicated that GCs can affect hippocampal neurogenesis. Martínez et al.^48^ have demonstrated that moderately elevated circulating CORT levels dramatically increase the number of newborn cells in the adult hippocampal DG. However, persistently high levels of plasmatic GCs can suppress adult hippocampal neurogenesis^49^ and promote the onset of neurodegenerative diseases.^50^ Prior research has also shown that while high levels of GCs mediated by chronic stress can also generate LTD and impair spatial memory,^25^ modest levels of GCs can enhance LTP and increase spatial learning and memory capacity.^24^, ^51^ Research has further shown that GCs regulate hippocampal plasticity mainly through two intracellular receptors, MR and GR.^33^ The hippocampal DG is one of the regions with the highest abundances of both GR and MR expressions in the brain.^52^, ^53^ Previous evidence has shown that MR has a high affinity for GCs which is activated even at physiological levels, whereas GR can only be activated in situations when there is an elevated level of GCs, such as during the zenith of the circadian rhythm or in response to stress. Adult mice with genetic knockdown of MR have fewer granule cells and neurogenesis in the hippocampus.^35^ Moreover, selective silencing of GR in newly formed hippocampal cells speeds up the process of neuronal differentiation and migration.^54^ In this study, we found that physiological levels of GCs could promote the proliferation of NSCs and adult hippocampal neurogenesis. These effects may be related to MR activation. However, stressful levels of GCs could decrease the proliferation of NSCs and adult hippocampal neurogenesis by activating GR, and may even lead to the apoptosis of NSCs.

MR and GR are also members of the ligand‐inducible transcription factor superfamily, which form multiprotein complexes with molecular chaperone proteins (such as Hsp70 and Hsp90) in the cytoplasm.^55^, ^56^ Once binding with GCs, MR or GR is released from the molecular chaperone complexes, migrates into nucleus, and then engages in GC‐responsive elements to regulate gene expression.^57^, ^58^ Further studies have found that the release of GC‐receptor complexes can also directly play with several kinds of transcription factors (NF‐κB, STAT3, AP‐1, etc.) to regulate gene expression.^59^, ^60^ In this study, we found that physiological and stressful levels of GCs could bidirectionally modulate TERT expression by regulating MR and GR, respectively. TERT, the catalytic component of telomerase, is essential in regulating telomerase activity and preserving telomere integrity.^61^ In addition, TERT also has a significant role in promoting neuronal growth and the development of the brain.^62^, ^63^ Fu et al.'s^64^ studies reported that brain‐derived neurotrophic factor (BDNF) increases TERT expression in embryonic hippocampal neurons. Conversely, inhibiting the production of TERT abolishes the effect of BDNF on neuronal survival. Moreover, mice lacking TERT displayed noticeable changes in anxiety‐related behaviors.^65^ Our earlier research also identified that hippocampal TERT was involved in the regulation of mood‐related behaviors through regulating NPC proliferation.^18^ Furthermore, the function of TERT in NPCs in controlling brain growth is independent of telomerase enzyme activity. This suggests that creating TERT that lacks catalytic activity could potentially enhance the formation of memories without the risk of tumor formation.^19^ In recent years, studies have also found that GCs may increase the survival probability of gull hatchlings by enhancing the activity of telomerase and prolonging the length of telomere.^36^ In this work, we also discovered that TERT overexpression could reverse the decline in learning and memory, as well as depressive symptoms, caused by elevated GC levels or GC deprivation in mice.

Although the detrimental impacts of GCs on cell proliferation in the hippocampal DG have been well described, it has also been observed that increased levels of GCs can promote neuronal growth in certain circumstances.^66^ For example, hippocampal DG progenitor cells are protected from the deleterious effects of high levels of GCs during reward experiences. Additionally, sexual experiences could also promote adult hippocampal neurogenesis, despite an initial elevation in CORT.^67^ This paradoxical effect of GCs may be related to cell and tissue specificity, such as the local availability of CORT and the expression of receptor variants. Nevertheless, the fundamental mechanism remains to be not well described in the present study. Further research is needed in this area.

In summary, this current study has identified that the bidirectional regulation of TERT by different GC concentrations is a key mechanism mediating the U‐shaped effects of GCs in the brain. Replenishment of TERT could be a common treatment strategy for GCs dysfunction‐associated psychiatric diseases.

## AUTHOR CONTRIBUTIONS

Meng‐Ying Liu, Yixin Fan, and Ningjie Ni performed western blotting and analyzed the results. Meng‐Ying Liu, Yixin Fan, Tao Yu, Zhiyuan Mao, and Hanyu Huang performed the animal experiments, Fluoro‐Jade staining, and flow cytometry. Jing Zhang, Fan Meng, Yulin Tang, and Hongliang He carried out the cell cultures and immunofluorescence assay. Meng‐Ying Liu, Yixin Fan, Yongping You, and Qi‐Gang Zhou conceived the study, participated in its design and coordination, and helped to draft the manuscript. Fan Meng, Yongping You, and Qi‐Gang Zhou helped revise the manuscript. All the authors approved the final manuscript. Meng‐Ying Liu, Yixin Fan, Ningjie Ni, and Tao Yu contributed equally to the study. All authors read and approved the final manuscript.

## CONFLICT OF INTEREST STATEMENT

The authors do not have any conflicts of interest to disclose.

## Supporting information


Data S1.

## Data Availability

Data can be provided upon request.
