# Supplementary material for: TERT mediates the U‐shape of glucocorticoids effects in modulation of hippocampal neural stem cells and associated brain function
Source: CNS Neurosci Ther. 2024 Feb 7;30(2):e14577. doi: 10.1111/cns.14577 (PMC10850922; doi:10.1111/cns.14577)
Supplement: Supplementary file 1 — Data S1. [file CNS-30-e14577-s001.pdf]

Figure 3A

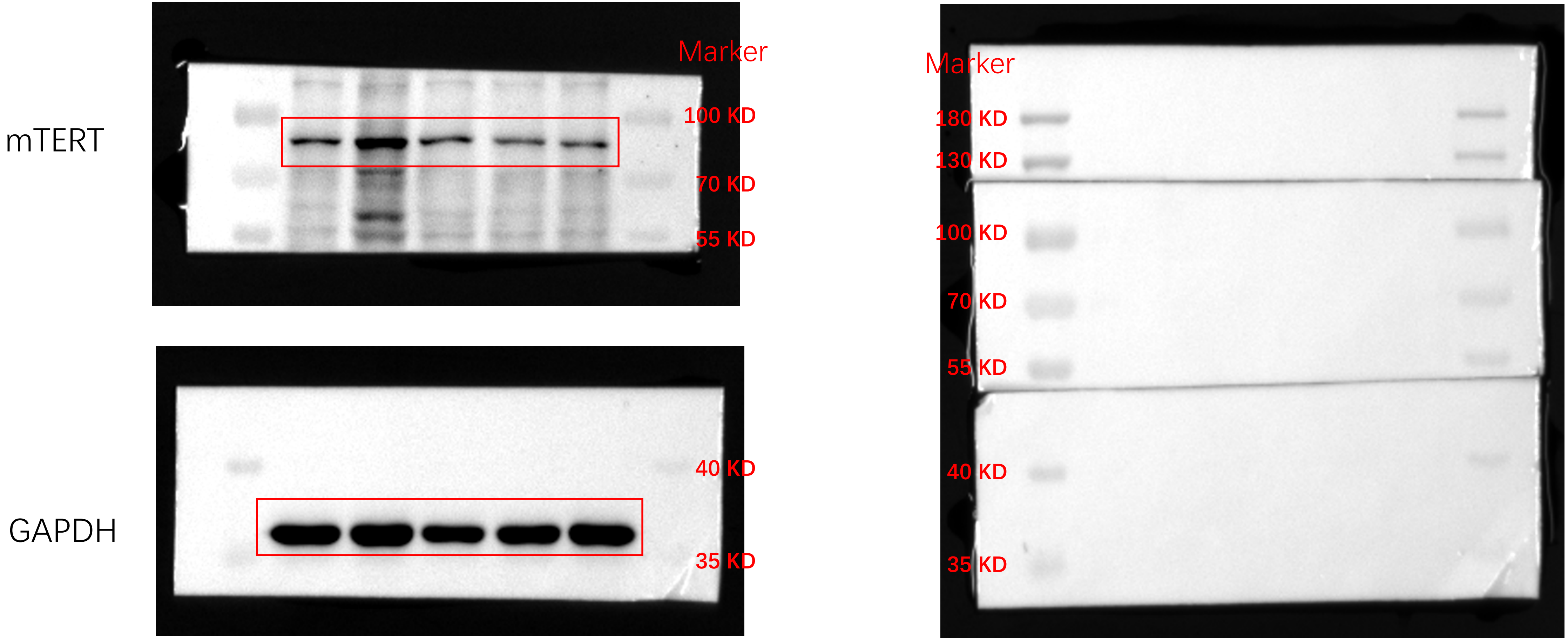

Left: The target protein band

Right: whole protein membrane

Figure 4C

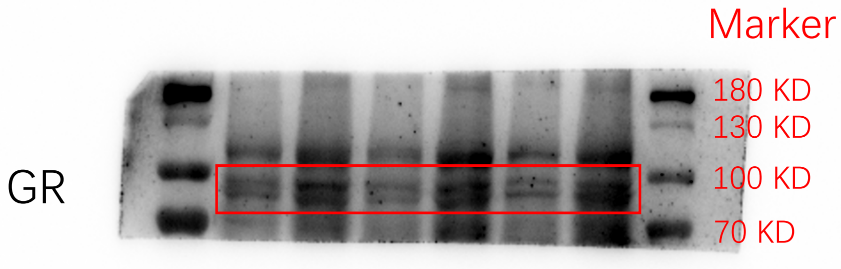

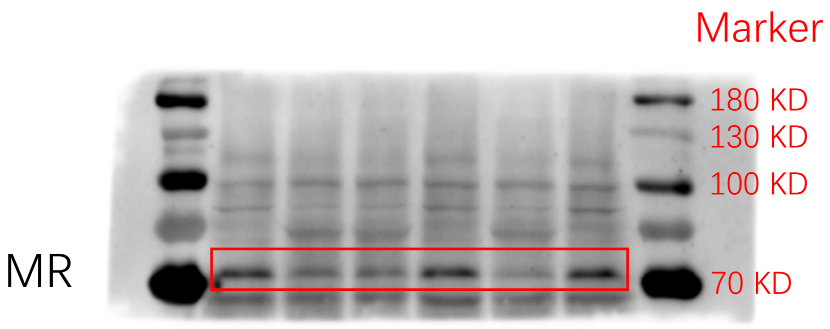

Left: The target protein (GR) band

Left: The target protein (MR) band

Figure 4C

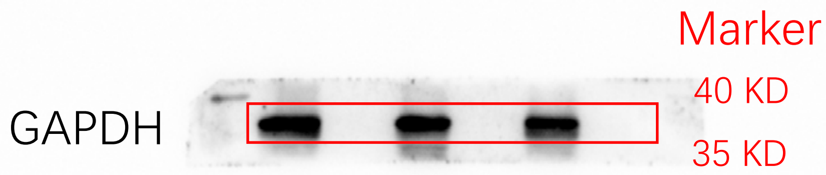

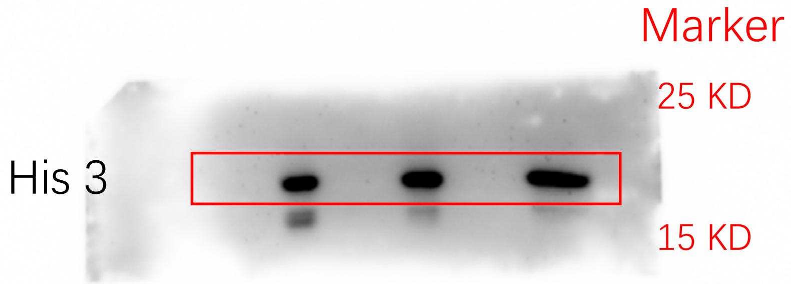

Left: The target protein (GAPDH) band

Left: The target protein (His 3) band

Figure 4C

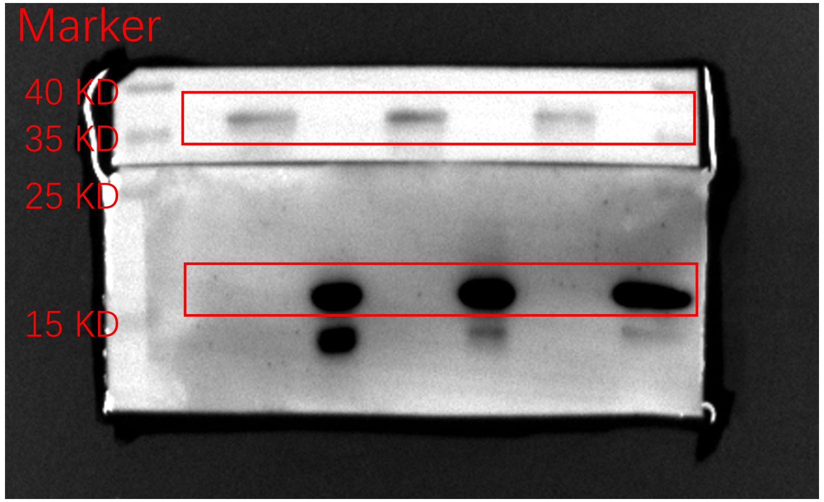

The whole protein membrane of GAPDH and His 3

Figure 4C

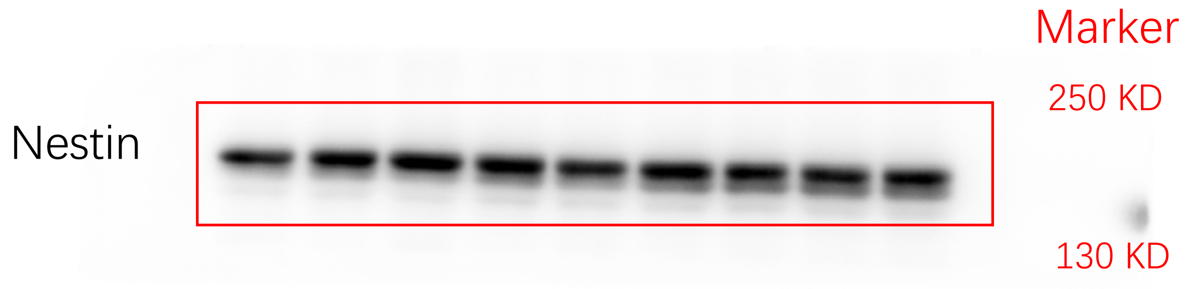

Left: The target protein (Nestin) band

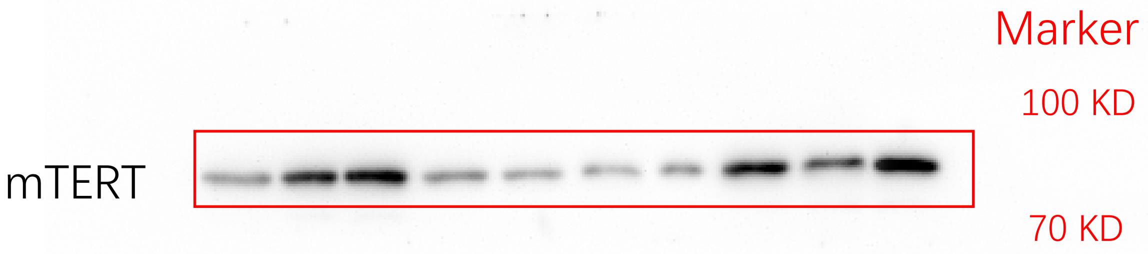

Left: The target protein (mTERT) band

Figure 4C

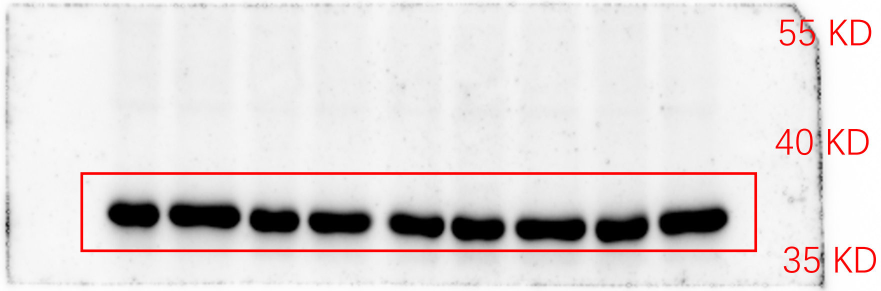

The band of GAPDH
